# Sensitivity and specificity in prevalence studies: The importance of considering uncertainty

**DOI:** 10.6061/clinics/2020/e2449

**Published:** 2020-11-26

**Authors:** Rafael Izbicki, Márcio A. Diniz, Leonardo S. Bastos

**Affiliations:** I Departamento de Estatisticas, Universidade Federal de Sao Carlos, Sao Carlos, SP, BR.; II Programa de Computacao Cientifica (PROCC), Fundacao Oswaldo Cruz, Rio de Janeiro, RJ, BR.

Serological surveys, such as EPICOVID19 (1), are important to monitor the evolution of COVID-19 in a population. In this letter, we discuss how to best estimate its prevalence. It is well known that the naive estimator of prevalence that consists of counting how many individuals tested positive ignores the possibility of test errors and may therefore substantially bias the conclusions. The often-used Rogan-Gladen estimator (2) is an alternative that provides corrected confidence intervals based on sensitivity/specificity values. However, this estimator has two main issues: (i) it often yields negative estimates of prevalence, and (ii) it assumes that the precision of the test is known with certainty, which is never the case; sensitivity/specificity are estimated from data. In this letter we focus on (ii) and demonstrate that taking the uncertainty regarding the precision of the test into account provides a different perspective for serological surveys. Our illustrative example is based on ENE-COVID (3), which investigates the prevalence of COVID-19 in Spain.

Since we do not have access to the exact numbers, we assume that among the 61,075 individuals in the survey, 3,054 (5%) tested positive on the point-of-care test. We use the sensitivity/specificity values provided in the paper: 82.1% (69.6%-91.1%) and 100.0% (96.5%-100.0%), respectively. For the sake of simplicity, we ignore sampling weights. Figure 1 shows 95% confidence intervals using different approaches. The naive estimate (*i.e*., the proportion of individuals that tested positive) has a small interval with no intersection with the Rogan-Gladen estimate, which is also short. On the other hand, the Bayesian interval that takes this uncertainty (4) into account is much wider; it contains points that are consistent with very different stages of the evolution of the epidemic. It is evident that the interval should be wide; it is not possible to recover the prevalence of the disease from data about the proportion of positive tests alone. Indeed, the statistical model is not identifiable (5). We conclude that uncertainties must also be transparently reported to subsidize decisions properly. An app that performs the analyses presented on new data can be found at https://rizbicki.shinyapps.io/tests/.

## Figures and Tables

**Figure 1:**
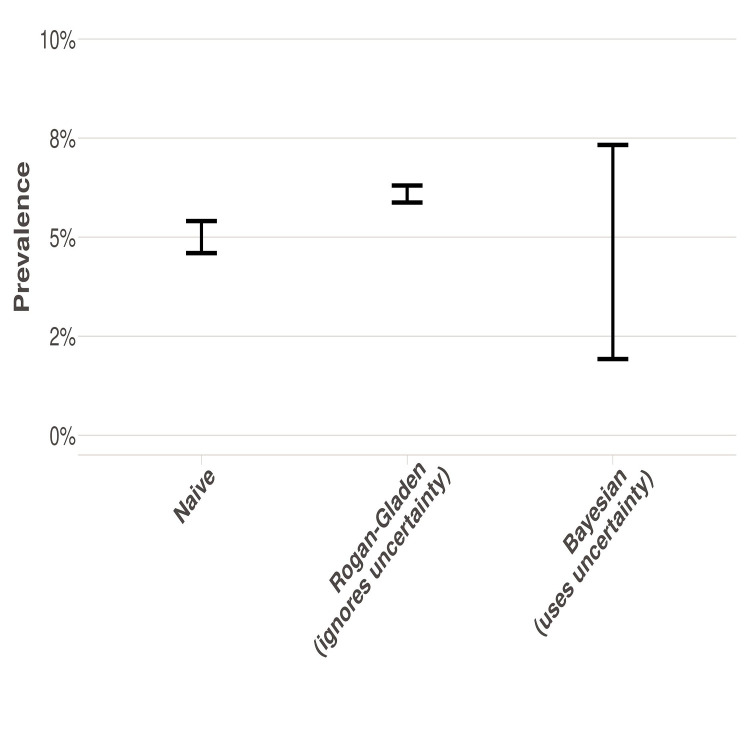
Confidence (Naive and Rogan-Gladen) and credible (Bayesian) intervals for prevalence of COVID-19.
